# Prevalence of Viral Hepatitis in Three Public Health Clinics in the Kingston Metropolitan Area, Jamaica, 2022

**DOI:** 10.4269/ajtmh.25-0359

**Published:** 2026-04-07

**Authors:** Jenene R. Cameron, Venise McIntosh-Morgan, Niel McKnight, Keisha Francis, Leshawn Mendoza, Abbas Hadji, Francisco Averhoff, Mary Rodgers, Gavin Cloherty, Susan Strachan-Johnson, John Lindo, J. Peter Figueroa, Michelle Brown, Joshua J. Anzinger

**Affiliations:** ^1^ The University of the West Indies, Department of Microbiology, Kingston, Jamaica, West Indies;; ^2^ Ministry of Health and Wellness, Kingston, Jamaica;; ^3^ Abbott Laboratories, Abbott Park, Illinois, USA;; ^4^ The University of the West Indies, Department of Community Health and Psychiatry, Kingston, Jamaica, West Indies;; ^5^ Abbott Pandemic Defense Coalition, Abbott Park, Illinois, USA;; ^6^ Abbott Pandemic Defense Coalition, Kingston Jamaica, West Indies;; ^7^ Global Virus Network, Tampa Bay, Florida, USA

## Abstract

Viral hepatitis is a global public health concern, yet surveillance data is lacking for many low-middle income countries, particularly in Jamaica where recent prevalence data is lacking. Serum samples were collected from patients attending antenatal (ANC; *n* = 530), non-communicable disease (NCD; *n* = 826) and sexually transmitted infection (STI; *n* = 517) clinics in the Kingston Metropolitan Area. All samples were tested for antibodies to hepatitis A – E viruses using the ABBOTT ARCHITECT to test for HAV IgG, HBV core total Ig, HCV total Ig, HDV total Ig, and HEV IgG, respectively, to assess for previous infection. The seroprevalence of HAV and HBV antibodies were ANC, 61.3%; NCD, 89.4%; STI, 65.4%; and ANC, 7.7%; NCD, 20.8%; STI, 17.2%; respectively, whilst HCV, HDV and HEV antibodies were all ∼1%. The seroprevalence of all types of viral hepatitis increased with age, notably for HAV and HBV. As HBV can cause chronic infections, HBV infection status was determined using the following tests: HBV surface antigen (HBsAg), HBV core IgM, HBV core total Ig, HBV surface antigen Ig. Of the patients testing HBV core total Ig positive, the prevalence of acute HBV infection was noted to be 2.5%, 0%, 0% and chronic HBV infection was 12.5%, 9.6% and 5.1% in the ANC, NCD and STI clinics, respectively. Collectively, our data indicate that public health measures should be focused on HAV and HBV infections in Jamaica, with more robust surveillance and targeted vaccination for populations at risk of infection to limit transmission.

## INTRODUCTION

The public health impact of viral hepatitis is substantial, with a worldwide prevalence of >300 million and incidence of 2.2 million per year reported in 2022.^1^^,^^2^ The number of deaths due to viral hepatitis has increased from 1.1 million (2019) to 1.3 million (2022), making it the second most common cause of death in 2022 from a communicable disease after COVID–19.^1^ Collectively, hepatitis A, B, C, D and E viruses (HAV, HBV, HCV, HDV, and HEV)^2^ are major contributors to liver disease, but their prevalence can vary greatly between countries. The World Health Organization (WHO) has highlighted the public health threat of viral hepatitis by implementing a global health strategy for the elimination of viral hepatitis by 2030.^3^ However, many low-to-middle income countries (LMICs) lack basic data to determine the prevalent types of viral hepatitis.

Hepatitis A virus and HEV represent the most common causes of acute viral hepatitis worldwide, though the prevalence can vary by geographical region. Their impact on public health is considerable, with 159 million^4^ and ∼20 million cases in 2019,^5^ respectively. Globally, the incidence of HAV increased from 140 million cases in 1990 to 159 million cases in 2019, 144 million (90.6%) of which were in LMICs.^6^ A study conducted in Jamaica >25 years ago showed that the overall prevalence of HAV was 60%.^7^ Approximately 20 million persons worldwide are infected with HEV per year, though this may be an underestimate due to the lack of prevalence information for many countries, the close resemblance to other hepatitis related conditions and the lack of diagnostic tests available.^8^ Hepatitis E virus is most prevalent in LMICs, particularly in many African countries, Mexico and South and South-east Asian countries, with high prevalence noted in some high-income countries (HICs) such as France, Poland, and Denmark.^9^^,^^10^ The overall prevalence of HEV in the Jamaican population was reported to be 2.1% in a small study of antenatal patients >10 years ago.^11^ Hepatitis A virus and HEV have become problematic because of their association with foodborne outbreaks, particularly in non-endemic settings where imported ready-to-eat foods such as fruits have been implicated in HAV outbreaks and contact or ingestion of contaminated or infected meats such as wild boar and pigs have been implicated in HEV outbreaks.^12^

Hepatitis B virus is a substantial public health burden, with ∼1.1 million deaths worldwide each year, with most noted in LMICs.^1^^,^^13^ Deaths are primarily due to liver failure, cirrhosis, and hepatocellular carcinoma resulting from chronic infection.^1^ In 2022 there was an HBV incidence of 1.23 million per year and an estimated 254 million persons with active HBV infection.^1^ All previous HBV surface antigen (HBsAg) prevalence studies from Jamaica were conducted >20 years ago in varying at risk groups and ranged from 3.2% to 15%.^14^^–^^16^ Hepatitis B virus is also known to play an active role in HDV infection as HDV replication can only occur with HBV infection.^17^ The seroprevalence of HDV across different Global Burden of Diseases regions in the general population ranged from 0.0% to 4.0%, and in individuals who were HBsAg carriers, ranged from 0.2% to 15.4%.^17^ An estimated 12 million persons are infected with HDV worldwide,^18^ but due to a significant lack of HDV surveillance data globally this number may be an underestimate. Hepatitis D virus prevalence was investigated in a sample of hemodialysis patients in Jamaica >10 years ago, but no positive patients were identified.^19^ The transmission of HDV is like that of HBV,^2^ with low infection rates reported in Northern Europe and the United States and high infection rates in the Mediterranean basin associated with injection drug use.^17^^,^^20^

Like HBV, HCV can cause chronic infection and life-threatening liver disease. Acute HCV infection is commonly asymptomatic; however, acute infections can progress to chronic disease that can lead to liver failure, cirrhosis, hepatocellular carcinoma and possibly death in millions of persons worldwide.^1^ Approximately 50 million persons are infected with HCV, with an incidence of 980,000, and approximately 218,000 deaths each year.^1^ Countries with a high reported prevalence of HCV include the Eastern Mediterranean Region: Egypt, Somalia, Sudan and South-east Asia and the Western Pacific Region.^2^ Groups at highest risk of HCV infection according to Centers for Disease Control and Prevention (CDC) surveillance data include persons who inject drugs (PWID), surgical procedures, multiple sexual partners and dialysis patients.^21^ The prevalence of HCV infection in Jamaica was investigated >20 years ago in patients who often received transfusions, including hemophiliacs (41%)^22^ and sickle cell disease patients (2%).^23^

The prevalence of the different types of viral hepatitis is unknown for many countries in the Caribbean region and existing data is largely dated. In Jamaica, the most recent viral hepatitis prevalence studies were all performed over a decade ago, the majority with small sample sizes of less than 1000. In this study we evaluated the recent prevalence of viral hepatitis by performing a cross-sectional serosurvey of viral hepatitis (A-E) in the Kingston Metropolitan Area amongst 1873 participants attending antenatal, non-communicable disease, and sexually transmitted infection public health clinics in 2022.

## MATERIALS AND METHODS

### Study design.

Approximately 1,873 stored serum samples collected between March 16, 2022 and May 5, 2022 for a study conducted in Kingston to assess the seroprevalence of COVID–19 antibodies were used.^24^ Samples were collected from nearly all patients attending the antenatal (*n* = 530) and sexually transmitted infection (STI, *n* = 517) clinics and the vast majority of patients attending non-communicable diseases (NCD, *n* = 826) clinics at health centers across the Kingston Metropolitan Area (Table 1). The clinics were targeted to represent the adult population of the Kingston Metropolitan Area by including both sexes, pregnant women, and a broad range of ages. NCD and STI clinic participants were 25.5% and 39.3% male, respectively. This sex distribution is likely related to the better health seeking behavior of women compared to men.^25^^,^^26^ Serum samples were stored at −20° C and testing began in November 2023.

**Table 1:** Sociodemographic profile of the participants

Variable	ANC, *n*^ (%)	NCD, *n* (%)	STI, *n* (%)	All Clinics, *N* (%)
Gender				
Male	0 (0.0)	194 (23.5)	232 (44.9)	426 (22.7)
Female	530 (100.0)	632 (76.5)	285 (55.1)	1,447 (77.3)
Total	530 (100.0)	826 (100.0)	517 (100.0)	1,873 (100.0)
Age group				
<20*	63 (11.9)	7 (0.9)	78 (15.1)	148 (7.9)
20–29	311 (58.9)	25 (3.0)	180 (35.0)	516 (27.7)
30–39	142 (26.9)	30 (3.6)	92 (17.8)	264 (14.1)
40–49	12 (2.3)	86 (10.5)	76 (14.7)	174 (9.3)
50–59	0 (0.0)	227 (27.6)	52 (10.1)	279 (15.0)
60–69	0 (0.0)	240 (29.2)	26 (5.0)	266 (14.3)
>70	0 (0.0)	207 (25.2)	12 (2.3)	219 (11.7)
Total	528 (100.0)	822 (100.0)	516 (100.0)	1,866 (100.0)

* The youngest age included in the study was 10 years of age.

^ *n* = number.

Sample sizes were adequate to compare frequencies for each of the three groups given a confidence level of 95% using OpenEpi, Version 3, open source calculator - SSPropor.^27^

### Laboratory testing.

All tests were by chemiluminescence immunoassay (CMIA) using an Abbott ARCHITECT *i*2000SR. The following tests were used to determine previous exposure: HAV IgG for HAV, HBV core total Ig for HBV, HCV total Ig for HCV, HDV Total Ig RUO for HDV, and HEV IgG RUO for HEV. For all tests the manufacturer’s recommended cutoff was used. Hepatitis D virus testing was only done for those positive by HBV core total Ig testing. To determine HBV infection status the following supplemental testing was performed: HBsAg, HBV core IgM and HBV surface antigen Ig. Acute hepatitis B infection was defined as HBsAg positive, HBV core IgM positive and HBV core total Ig positive. Chronic hepatitis B infection was defined as HBsAg positive, HBV core IgM negative and HBV core total Ig positive. HBV surface antigen Ig testing was not performed for individuals who tested positive for HBV core total Ig, positive for HBsAg, and negative for HBV core IgM, as these cases were presumed to represent chronic HBV infection. The design of this study was cross-sectional; therefore, it was not possible to have patients re-tested. Additional clinical information was not collected and was not included in the ethical approval. Resolved infection was defined as HBV core total Ig positive, HBsAg negative, and HBV core IgM negative; or HBV core total Ig positive, HBsAg negative, HBV core IgM positive and HBV surface antigen Ig positive. HBV surface antigen Ig testing was not done on all samples assessed as resolved infection due to many samples having insufficient volumes. Therefore, we could not have assessed the prevalence of occult hepatitis B virus infections. However, occult hepatitis B virus infections would be expected to be present only in a small number of infected persons, as the prevalence was previously shown in Western countries to be 0.1%–2.4% and in endemic areas the prevalence was 6%.^28^

## STATISTICAL ANALYSES

Data was analyzed using IBM SPSS Statistics for Windows version 20 (IBM Corp., Armonk, New York, USA) and represented in tabular forms and data was analyzed using GraphPad version 10.3.1 and represented in graphical forms. Both categorical and continuous variables were assessed using Pearsons and Fisher’s Exact Chi-square tests. A *P*-value of <0.05 was considered statistically significant.

## RESULTS

We first assessed the seroprevalence of HAV IgG, HBV core total Ig, HCV total Ig, HDV total Ig (only HBV positive samples tested), and HEV IgG amongst the three clinics (Table 2). Hepatitis D virus infection can only occur in the presence of HBV infection, so HDV prevalence was calculated for samples testing HBV positive. The seroprevalence of HCV, HDV and HEV was low (≤1.1%) amongst all clinics. In contrast, HAV seroprevalence was 61.3% for ANC, 89.4% for NCD, and 65.4% for STI. Hepatitis B virus seroprevalence was 7.7% for ANC, 21.1% for NCD, and 17.2% for STI. The seroprevalence of all types of viral hepatitis was greatest amongst the NCD clinic, the population with the oldest age, suggesting that viral hepatitis prevalence was associated with age.

**Table 2:** Overall prevalence of viral hepatitis infections

Variable	ANC		NCD		STI	
	Tested, *n*^	Positive, *n* (%)	Tested, *n*	Positive, *n* (%)	Tested, *n*	Positive, *n* (%)
Anti-HAV (IgG)	530	325 (61.3)	819	732 (89.4)	515	337 (65.4)
Anti-HBc (IgM and IgG)	530	41 (7.7)	821	171 (20.8)	516	89 (17.2)
Anti-HCV (IgM and IgG)	530	3 (0.6)	825	9 (1.1)	515	2 (0.4)
Anti-HDV* (total Ig)	41	0 (0.0)	158	2 (1.3)	82	0 (0.0)
Anti-HEV (IgG)	523	4 (0.8)	788	13 (1.6)	495	3 (0.6)

* Anti-HDV testing was only performed for samples testing positive for anti-HBc (IgM and IgG).

^ *n* = number.

**Figure 1.**
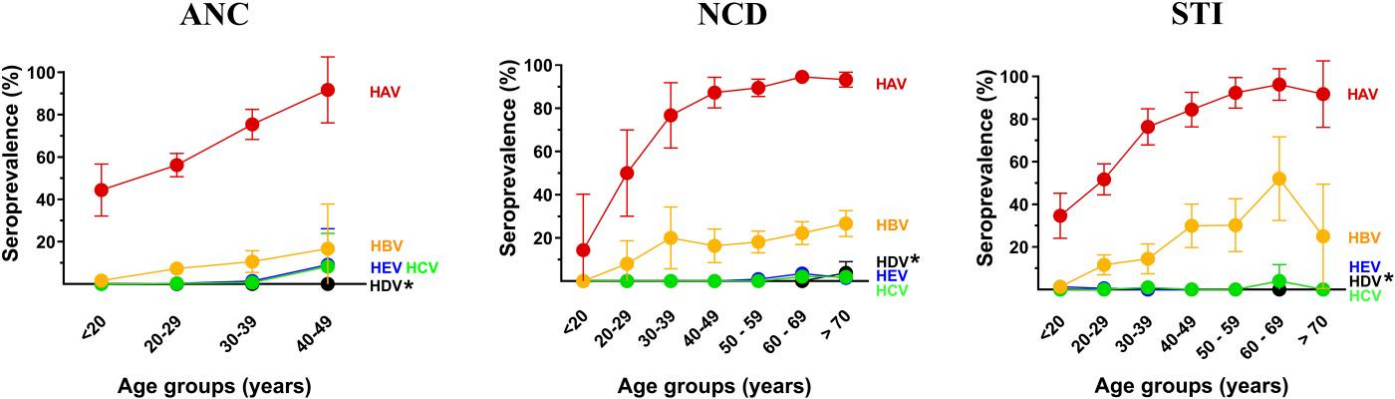
Prevalence of Viral Hepatitis by Age Groups. *Anti-HDV testing was only performed for samples testing positive for anti-HBc (IgM and IgG). HAV seroprevalence significantly increased with age in all clinics: ANC: *χ^2^* [3] = 27.270, *P* <0.001; NCD: *χ^2^* [6] = 95.580, *P* <0.001; STI: *χ^2^* [6] = 96.609, *P* <0.001. HBV seroprevalence increased with age in all clinics but was only significant for the STI clinic: ANC: *χ^2^* [3] = 6.373, *P* = 0.095; NCD: *χ^2^* [6] = 10.883, *P* = 0.092; STI: *χ^2^* [6] = 54.596, *P* = 0.001. Values represent mean ± standard deviations.

For each clinic, seroprevalence increased with age for HAV and HBV (Figure 1). The prevalence of HAV was noted to be >2-fold greater in the >40 years age group compared to the <20 years age group in each clinic, likely due to many infections occurring during adulthood. For HCV, HDV, and HEV, almost all samples testing positive were in the ≥60 years of age group in the NCD and STI clinics, and ≥40 years age group amongst the ANC clinic (Figure 1). Only two samples tested HDV positive in the NCD clinic. Amongst all participants, those testing HEV positive were also HAV positive. No association was observed between viral hepatitis prevalence and sex.

Hepatitis B virus, HCV and HDV can each progress to chronic infection which can lead to debilitating medical conditions such as liver failure, cirrhosis and hepatocellular carcinoma. We chose to further assess HBV types of infections as HBV was far more prevalent than HCV and HEV and we only had sufficient sample volume for HBV supplemental testing. The proportion of HBV infections that were active or resolved infections were determined for both those testing HBV core total Ig positive and from the total study population (HBV core total Ig positive and negative) (Table 3). A total of 301 samples were HBV core total Ig positive, however, only 275 samples were tested for HBsAg because 26 samples had insufficient volumes for supplemental testing. For those testing HBV core total Ig positive, 12.5%, 9.6% and 5.1% of persons had chronic HBV infection from the ANC, NCD and STI clinics, respectively, and only 1 person (2.5%) from the ANC clinic had acute HBV infection. When considering the total study population (HBV core total Ig positive and negative), 0.9%, 1.8% and 0.8% of persons had chronic HBV infection from the ANC, NCD and STI clinics respectively and only 1 person (0.2%) from the ANC clinic had acute HBV infection. It is possible that some chronic infections could be occult hepatitis B virus infections. Insufficient testing volumes prevented us from assessing this type of HBV infection; however, occult hepatitis B virus infections would only be expected to be present for a small fraction of HBV infected patients.^28^

**Table 3:** Prevalence of hepatitis B virus infection by infection status type

Infection Status	ANC		NCD		STI	
	Tested, *n*^	Infection, *n* (%)	Tested, *n*	Infection, *n* (%)	Tested, *n*	Infection, *n* (%)
HBV core total Ig positive						
Active^a^	40	6 (15.0)	156	15 (9.6)	79	4 (5.1)
Acute^b^	40	1 (2.5)	156	0 (0.0)	79	0 (0.0)
Chronic^c^	40	5 (12.5)	156	15 (9.6)	79	4 (5.1)
HBV core total Ig positive						
Resolved^d^	40	34 (85.0)	156	141 (90.4)	79	75 (94.9)
HBV core total Ig positive and negative^e^						
Active^a^	530	6 (1.1)	821	15 (1.8)	516	4 (0.8)
Acute^b^	530	1 (0.2)	821	0 (0.0)	516	0 (0.0)
Chronic^c^	530	5 (0.9)	821	15 (1.8)	516	4 (0.8)
HBV core total Ig positive and negative^e^						
Resolved^d^	530	34 (6.4)	821	141 (17.2)	516	75 (14.5)

^a^ Active – HBsAg positive.

^b^ Acute – HBV core total Ig positive, HBsAg positive and HBV core IgM positive.

^c^ Chronic – HBV core total Ig positive, HBsAg positive and HBV core IgM negative.

^d^ Resolved – Criteria 1 - HBV core total Ig positive, HBsAg negative, and HBV core IgM, negative. Criteria 2 - HBV core total Ig positive, HBsAg negative, HBV core IgM positive and HBV surface antigen Ig positive.

^e^ Only HBV core total Ig positive samples were tested for supplemental HBV markers.

^ *n* = number.

## DISCUSSION

This cross-sectional serosurvey, to our knowledge, is the first to assess all major types of viral hepatitis in Jamaica, is the largest reported and provides much needed current data. Importantly, our data show that most viral hepatitis in Jamaica is due to HAV and HBV, with only a very small minority of persons having serological evidence of having been infected with HCV, HDV, or HEV.

Hepatitis B virus can cause chronic infections that are transmissible and can lead to severe, potentially fatal disease. Our data shows that 15% (ANC), 9.6% (NCD) and 5.1% (STI) of HBV infections are active and hence capable of onward transmission. Almost all HBV infections were due to chronic infection (12.5%, ANC; 9.6%, NCD; and 5.1%, STI), in which 15 - 40% would be expected to develop cirrhosis, hepatocellular carcinoma (HCC) or liver failure.^29^ Molecular testing of patients with chronic HBV infection would be useful to determine viral loads which can assist with determining risk of progression to liver disease, the need for initiation of treatment^30^ and phylogenetic analysis to determine genotypes and dissemination. Although not done in this study, HBV phylogenetic analysis was previously assessed for University Hospital of the West Indies patients over the period 2019 – 2021, showing that 75% were genotype A1 and 25% were B2.^31^

The 17.2% HBV core total Ig and 0.8% HBsAg prevalence observed in the STI clinic for this study has decreased since 2001 when the prevalence was 21% for HBV core total Ig and 3.2% for HBsAg amongst patients attending the same clinic.^15^ This reduction in active infections among persons with STI encourages further steps to be taken towards elimination of the virus in this population. To our knowledge, the only other previous HBV prevalence studies were conducted in high-risk groups in Jamaica, with an HBsAg positivity of 15% in a sample of HIV-1 positive patients in 2002,^16^ and 5.3% HBsAg positivity in a sample of HCWs in 1991.^14^ Although the <2% HBsAg prevalence observed from each clinic in our study is considered a low endemicity setting,^32^ these active infections are concerning because of the lack of routine screening of persons at high risk in Jamaica and introduction of public childhood vaccination in 2003. The vaccination schedule at that time was a three-dose series (6 weeks, 3 months, 6 months) with a four-dose schedule introduced in 2023 (birth, 6 weeks, 3 months, 6 months). While this has led to a dramatic vaccine uptake in younger age groups (i.e. those persons born since 2003) many adults at risk of infection remain unprotected by vaccination.^33^ However, medical and nursing students in Jamaica have been vaccinated against HBV for decades.

The most likely HBV transmission routes in Jamaica are sexual transmission and mother-to-child transmission, as injection drug use (IDU)^34^ and transfusion related transmission are expected to be negligible in the population.^21^ The WHO has recommended that persons who are at high risk of contracting HBV infection such as multiply transfused persons, dialysis patients, men who have sex with men (MSM) and other groups should be vaccinated.^35^ This recommendation would be useful to implement for the at-risk Jamaican population born prior to 2003 in which HBV vaccination coverage is low. More public awareness and education are necessary for the adult population who have never been exposed or vaccinated, particularly those at highest risk. Notification of partners and household members of blood donors who are found to be infected during routine screening is one method used to increase HBV vaccine uptake in the adult population to limit onward transmission. Even though the HBV vaccine is routinely available in the public health system, it is offered at a cost to adults which may serve as a deterrent for vaccine uptake.

The adoption of HBV vaccination into the Jamaican national vaccination schedule in 2003 is expected to lead to dramatic reductions in HBV infection in young persons, as shown from modeling of the limited available data from Jamaica.^13^ In our study very few persons in the <20 years age group showed serological evidence of HBV infection, but it is uncertain if this is due to the introduction of HBV public vaccination in 2003 and/or that transmission is lower in this age group in the absence of vaccination. All active HBV infections in the antenatal population were in the ≥20 years age group. These active infections are concerning due to the increased risk of perinatal transmission.^30^ The recent introduction of the HBV vaccine birth dose in the national immunization schedule is one method that has been implemented to reduce mother-to-child transmission (MTCT) in Jamaica which was previously shown to be 75% effective in reducing HBV MTCT in antenatal patients who were HBsAg positive.^36^ Introduction of the HBV birth dose has been one key strategy outlined in the WHO elimination plan of viral hepatitis by 2030, including routine screening of antenatal patients.^3^ In Jamaica, however, HBV screening is not done at routine antenatal visits and although the vaccine in the newborn greatly reduces the risk of MTCT it does not entirely prevent transmission. Therefore, institution of routine screening for HBV infection in antenatal patients would allow for identification of infected mothers in which MTCT could be further reduced with hepatitis B immune globulin (HBIG) until majority of the population is immune. HBIG in combination with HBV vaccine within 24 hours of birth reduces MTCT by 94% (HBIG has been shown to be 71% effective when given alone).^36^ Since HBV public vaccination was only recently introduced in Jamaica, future seroprevalence studies across age groups would be informative to determine if decreased transmission in younger age groups is occurring as expected.

Our data show the overall prevalence of HAV in the study was >60% in each clinic with 70% of the population being infected by 30 years of age and <50% of the <20 years age group having been infected, very similar to the prevalence reported during 1995–1998 when 73% of the population was infected by 30 years of age and 30% of the population was infected by 10 years of age,^7^ suggesting that prevalence across ages has remained largely the same. These data indicate that Jamaica remains a low HAV endemicity setting according to CDC criteria (seroprevalence of ≥50% by age 30 with <50% seroprevalence by age 15),^37^ a setting that is typically associated with increased risk of outbreaks and infection in older populations. These results could be explained by differences in exposure across ages but also could be due to an age-cohort effect related to improvements in public health measures with time and are both possible explanations for the increasing HBV prevalence with age. Molecular testing would assist with providing further information into the likely source of HAV infections in the country and the relatedness amongst the samples. Although Jamaica is a LMIC, the HAV endemicity closely resembles HICs^4^ but the explanation for this is uncertain. In HICs, where most persons are exposed in adulthood, the use of vaccinations and implementation of food safety protocols assists in the reduction of HAV cases^6^ and these strategies may be considered to limit the risk of outbreaks in Jamaica. Universal mass vaccination (UMV) programs with HAV vaccine, especially in children had shown a reduction of HAV infection in both endemic and non-endemic countries even amongst non-vaccinated persons due to herd immunity.^38^ Recommendation has also been given by WHO to vaccinate persons belonging to high-risk groups in areas of low and very low endemicity.^38^ Therefore, it may be beneficial to introduce HAV childhood vaccination or vaccination of high-risk groups to reduce the prevalence of HAV in older age groups. Although HAV vaccines are available in Jamaica, they are not included in the national immunization schedule and are associated with a fee. In addition to these problems, it may be difficult to identify persons at risk.

Hepatitis E virus transmission has not been extensively studied in the Jamaican and Caribbean population and with the poor availability of diagnostic methods, surveillance and detection is not commonly practiced in the region. The concern with HEV infection worldwide is its association with foodborne outbreaks in areas such as Europe and South-East Asia due to ingestion of raw or undercooked meats^9^^,^^39^ and waterborne transmission in many African and Asian countries due to poor sanitation.^39^ Foodborne and waterborne transmission of HEV do not appear to be problematic in the Jamaican population due to the low prevalence noted of <2% from each clinic for HEV in this study, far below the worldwide prevalence of 12.5%^40^ and lower than the prevalence reported in the neighboring United States population at 6.1%.^41^ Hepatitis E virus has only previously been investigated in Jamaica once in 2011 in a very small population of pregnant women that also reported a low seroprevalence of 2.1%.^11^ The seroprevalence of HEV in our study was noted to be increased in persons ≥50 years amongst each clinic which suggest more opportunity for exposure or the possibility of outbreaks in the distant past. Foodborne transmission is unlikely due to the methods of cooking used in Jamaica that allows for meats to be thoroughly cooked; however, waterborne transmission could have been possible in the past that would have gone undetected due to the co-circulation of HAV at the time. Although rare, HEV can be transmitted via blood transfusions,^42^ another possible mode of transmission in Jamaica as blood products are not routinely screened for HEV.

The prevalence in our study for HCV (∼1.0%) from each clinic and HDV (0.11% of the total study population) in the NCD clinic was below worldwide prevalences of 1.6%^43^ and 0.16% of the general population,^18^ respectively. Previous studies done >20 years ago in Jamaica showed that the prevalence of HCV amongst blood donors was, similar to our study, low at 0.3–0.4%.^23^ Hepatitis D virus was previously investigated in 63 hemodialysis patients in 1998; however, no positivity was found.^19^ Only 2 samples were anti-HDV positive in the current study and we previously did not identify any anti-HDV positive samples from 52 residual diagnostic University Hospital of the West Indies samples testing HBsAg positive (unpublished observation), indicating that HDV prevalence in Jamaica is likely very low.

The seroprevalence of HCV was increased in persons ≥40 years amongst each clinic. Injection drug use (IDU) is a well-known risk factor for HCV and HDV infection.^1^ It is unlikely that possible exposure to HCV and HDV in the study population could be attributed to IDU due to the low prevalence of this practice in Jamaica^34^ and a more probable consideration is exposure during blood transfusions, consistent with widespread screening of blood and blood products for HCV that was introduced in 1990.^44^ A study of hemophiliac patients conducted in 2002 in Jamaica showed an HCV prevalence of 41%, with a requirement for transfusion identified to be a potential risk factor for infection^22^ and in another study conducted in 1995 in sickle cell patients who received multiple transfusions, the HCV prevalence was reported to be >7 times that of the control group.^23^ Therefore, persons ≥40 years could have been exposed prior to the introduction of routine screening of blood and blood products in Jamaica. Implementation of routine HCV blood screening in Jamaica will likely lead to a reduction in prevalence over time, aligning with the WHO goal of eliminating HCV.

One of the main strengths of this study is that it is, to our knowledge, the largest viral hepatitis prevalence study that has been conducted in Jamaica thus far. Our stratification results by age groups allowed us to show that HAV and HBV infections were primarily in persons >40 years, indicating that this age group may be a useful target for vaccination as HAV infection can cause severe illness in older populations and HBV vaccination would not have been included in the national vaccination schedule for this population. Limitations of our study include the cross-sectional design which prevented follow up and re-testing of patients who were positive, and that molecular testing was not done to further confirm chronic HBV infections and to determine phylogenetic analysis.

## CONCLUSION

We show that amongst the hepatitis viruses, HAV and HBV infections are most common in Jamaica, with very low prevalence of HCV, HDV, and HEV infections. Our study was designed to provide an initial overview of the seroprevalence of viral hepatitis in the Kingston Metropolitan Area and so provides important information most relevant to public health stakeholders. Highly effective vaccines exist for both HAV and HBV that could be used more widely to further reduce transmission of these viruses in Jamaica. Future studies would be useful to assess possible risk factors and other clinical data associated with the development of liver disease in patients identified as having chronic hepatitis infection.
